# Carbon Interlayer with Uniformly Anchored ZnO Nanoparticles: Surface‐Energy‐Driven Coble Creep for Practical Anode‐Free Solid‐State Batteries

**DOI:** 10.1002/advs.202600057

**Published:** 2026-06-03

**Authors:** Joonhyeok Park, Jeongheon Kim, Seungwoo Lee, Jaeik Kim, Il Woo Ock, Seunggun Choi, Jooheon Sun, Gunwoo Cha, Seungmin Han, Hyungjun Lee, Jun Lim, Hyun‐Suk Kang, Jiung Cho, Jong Sung Jin, Seho Sun, Ungyu Paik, Taeseup Song

**Affiliations:** ^1^ Department of Energy Engineering Hanyang University Seoul Republic of Korea; ^2^ Department of Battery Engineering Hanyang University Seoul Republic of Korea; ^3^ Korens RTX R&D Center Gyeryong Republic of Korea; ^4^ Department of Materials Science and Engineering Hongik University Sejong Republic of Korea; ^5^ Busan Center, Korea Basic Science Institute Busan Republic of Korea; ^6^ School of Chemical Engineering Yeungnam University Gyeongsan Republic of Korea

**Keywords:** all‐solid‐state batteries, anode‐free, creep behavior, interfaces, sulfide‐based solid electrolytes

## Abstract

Anode‐free solid‐state batteries (AFSSBs) promise high energy density and improved safety, but their material/manufacturing costs and electrochemical performance remain challenging. Here, cost‐effective and high‐energy‐density AFSSBs are demonstrated using a zinc oxide–carbon composite interlayer (ZnO@C) synthesized via an electron‐beam (e‐beam) irradiation method. Strong chemical anchoring of ZnO nanoparticles (NPs) smaller than 5 nm on the carbon host ensures their homogeneous dispersion across the interlayer. These ZnO NPs lower the energy barrier for reacting with lithium and serve as a buffer layer during lithium deposition. Moreover, the ZnO NPs with high surface energy induce the formation of finer lithium nuclei, which improves the creep behavior. By suppressing nanoparticle agglomeration, the chemical anchoring preserves the nanoscale morphology during cycling. As a result, the ZnO@C layer anodes exhibit high energy density, stable Coulombic efficiency of greater than 99.8%, and cycle retention of 69.6% after 300 cycles.

## Graphical abstract

We used a zinc oxide–carbon composite interlayer (ZnO@C) via the electron‐beam (e‐beam) irradiation method to fabricate a cost‐effective, high‐energy‐density, anode‐free solid‐state battery. Chemical anchoring between the ZnO and carbon enabled uniform nanoscale morphology, mitigating the high surface energy of the ZnO NPs. This structure enhances lithium deposition, ensuring stable electrochemical performance and improved battery efficiency.

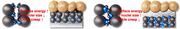

## 1 Introduction

Anode‐free solid‐state batteries (AFSSBs) are a promising energy‐storage technology due to their potential for higher gravimetric and volumetric energy densities compared with lithium metal batteries and lithium metal solid‐state batteries [1, 2]. In AFSSBs, the anode consists solely of a current collector without lithium metal, while the cathode serves as the only source of lithium during charging, thereby reducing the cell mass and thickness [3, 4, 5]. However, several challenges involving limited lithium sources have prevented widespread application of AFSSBs [6, 7]. The lithium in the cathode is the sole source of lithium, which is depleted due to the formation of the solid electrolyte interphase, repeated side reactions with the solid electrolyte (SE), and dead lithium. The absence of supplementary lithium to compensate for these irreversible losses results in low Coulombic efficiency (CE), rapid capacity degradation, and a reduced lifespan [8, 9, 10, 11, 12].

Numerous studies have attempted to address these issues. In 2020, Lee et al. from Samsung Electronics reported that using silver‐carbon nanocomposite layers could maintain a CE of greater than 99.8% for 1000 cycles [10]. This layer facilitates the formation of dense and uniform lithium deposits. The deposited lithium is formed underneath the silver‐carbon interlayer, which prevents physical contact between lithium metal and solid electrolytes, minimizing side reactions. Recent follow‐up studies have shown that silver maintains low interface resistance when reacting with lithium, which promotes uniform current distribution within the whole electrode [13, 14, 15, 16, 17, 18]. During deposition, lithium nanoparticles (NPs) move toward the underside of the interlayer (to the current collector) through Coble creep at the external high pressure of the AFSSB system [19, 20, 21, 22]. This composite of silver and amorphous carbon can significantly enhance the nucleation, growth, and migration of lithium, effectively eliminating dendritic growth and improving electrode stability. Previous studies have shown that this approach offers a promising pathway for developing more reliable AFSSBs [23, 24, 25, 26].

Despite the various advantages of a silver‐carbon layer, silver NPs are not practical for AFSSBs due to the high cost of the raw material. To address these challenges, significant efforts have been made in the use of alternatives to silver. Lithiophilic metals such as magnesium [27, 28, 29], tellurium [30, 31], antimony [32, 33], silicon [34, 35], and zinc [36, 37, 38, 39] are potentially cost‐effective alternatives that can reduce lithium‐nucleation overpotential. However, although a wide range of lithiophilic metal candidates has been investigated, high lithiophilicity alone has not guaranteed superior cell performance, and achieving performance surpassing that of silver has proved difficult. Recent studies report that the alloying reaction potential between lithiophilic metal and lithium critically influences lithium dissolution from the alloying matrix and the Coble creep behavior of nano lithium particles. Integrated computational–experimental studies on Ag/C buffer layers have reported that the alloying reaction voltage (a positive lithiation potential) provides the electrochemical driving force for lithium ingress and, when coupled with alloy‐induced volume expansion, governs the extrusion and creep‐like flow of the Ag–Li solid solution phase [40]. Among the lithiophilic candidates Sn, In, and Zn, Zn exhibits a reaction potential most similar to that of silver and, owing to its favorable mixed ionic–electronic conduction (MIEC), facilitates smoother creep behavior (Note S1) [38, 39].

Despite exhibiting electrochemical behavior and Li‑alloy formation similar to silver, zinc shows a higher interfacial reaction resistance and higher charge‑transfer resistance than silver. Density functional theory (DFT) calculations suggest that the relatively higher lithium diffusion barrier energy of zinc (6.458 eV) compared to silver (4.807 eV) may account for its slightly lower electrochemical performance (Note S2). This diffusion‑barrier issue indicates that the energy required for the initial insertion of lithium into the zinc lattice and for subsequent diffusion within zinc is higher than in silver. To overcome these limitations, it is necessary to stabilize the reactivity between zinc metals and lithium by modifying their surface properties and controlling their dimensions and particle sizes. Among these strategies, the most practical approach is to design alternative metals at the nanoscale to increase their specific surface area and enhance surface energy. In the reaction coordinates, the surface energy of the domain highly affects the kinetic favorability of lithium insertion into the lithiophilic domain (Figure S1). Moreover, the Gibbs–Thomson relation, according to Equation (1)

(1) $$\mu =\frac{2\gamma V_{m}}{r}$$

where µ, γ, V_m_, and *r* represent the chemical potential, surface energy, atomic volume, and particle radius, respectively, linking curvature to chemical potential and providing a capillarity‐driven force for mass transport at the nanoscale [41, 42]. However, even with an initially nanoscale morphology, Ostwald ripening may occur during repeated cycling due to the thermodynamic instability of nanoparticles resulting from their high surface energy. During the repeated alloying and de‐alloying reaction, the small metal particles aggregate to lower their surface energy [43]. This phenomenon leads to the subsequent growth of larger lithiophilic metal‐lithium alloy domains, inducing non‐uniform lithium nucleation and plating behavior, increasing interfacial resistance, and ultimately resulting in a gradual decline in electrochemical performance.

**FIGURE 1:**
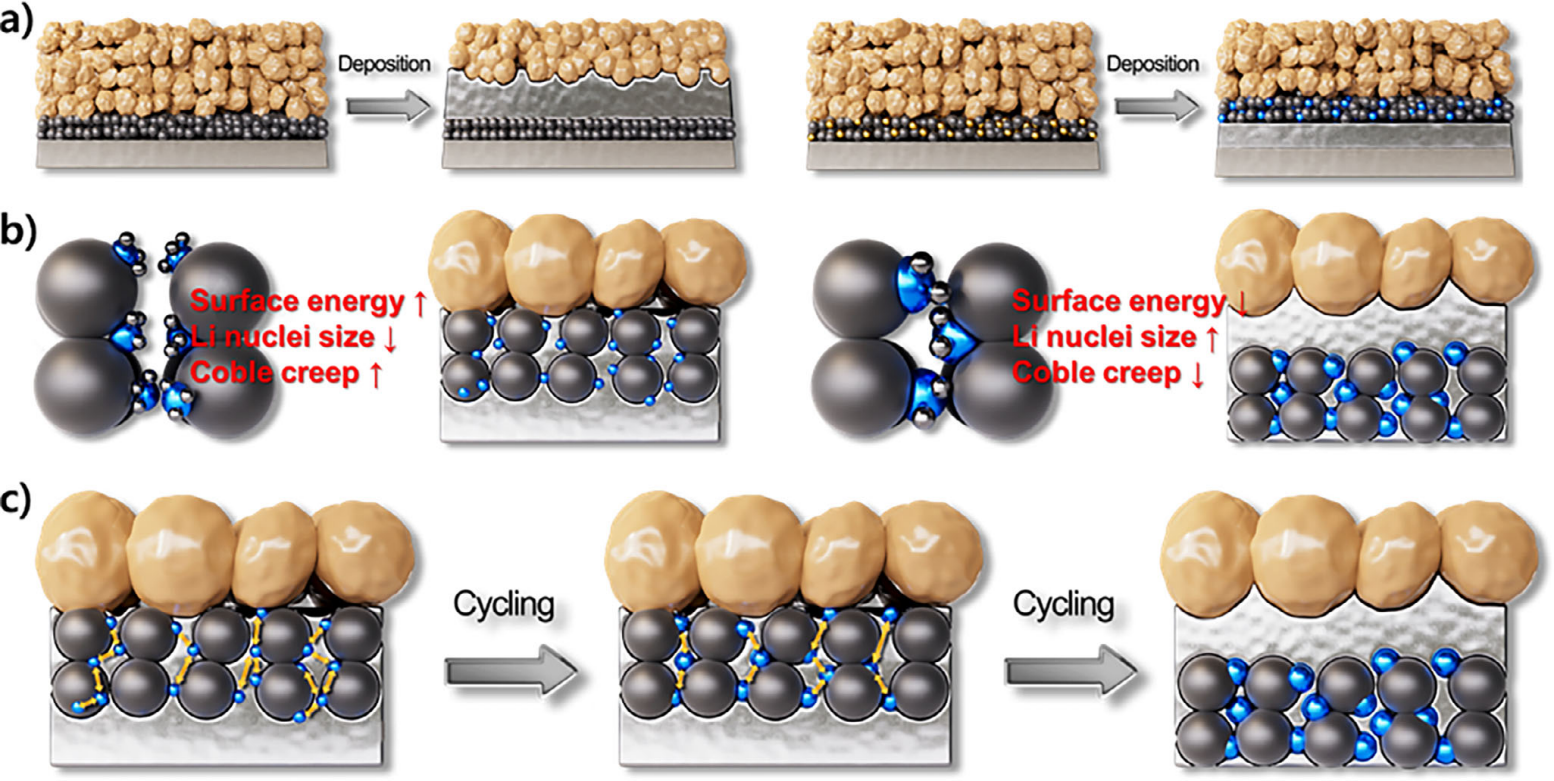
(a) Scheme of lithium deposition and the creep mechanism in a carbon layer and a carbon layer containing lithiophilic particles. (b) Scheme of a diffusion energy barrier for atoms in a lithiophilic metal with lithium during lithiation. (c) Scheme of creep mechanisms after lithium deposition across different lithiophilic particle sizes.

Figure 1a illustrates the lithium deposition behavior in AFSSBs on carbon interlayers with and without zinc incorporation. In the absence of zinc, lithium tends to deposit toward the solid electrolyte side rather than the current collector side (Figure S2) [19, 21]. This behavior is primarily attributed to the poor lithiophilicity and high nucleation barrier at the carbon interface without metal incorporation, which makes lithium nucleation less favorable at the current collector side. As a result, lithium tends to nucleate near the electrolyte interface, determining the initial growth direction and subsequently affecting the overall deposition behavior. Zinc serves as an effective nucleation site for lithium, directing uniform lithium deposition toward the current collector side, which suppresses side reactions at the electrolyte interface and enables stable lithium plating/stripping during cycling. As demonstrated by our DFT calculations, the particle size of zinc also plays a critical role in determining the lithium growth behavior (Figure 1b). The Gibbs free energy change of lithium nucleation (ΔG_Li_) for semicircular nuclei according to Equation (2)

(2) $$\Delta G_{Li}=-\frac{2}{3V_{m}}\pi r^{3}\Delta G_{B}+\pi r^{2}\sigma$$

where V_m_, *r* and σ represent the molar volume of the lithium, the radius of lithium nuclei and the surface tension at the interface, respectively, is related to the surface energy at the interface [44, 45]. To determine the radius of the critical nucleus (r*), ΔG_Li_ is differentiated with respect to radius (*r*), and by setting the derivative equal to zero, Equation (3)

(3) $$r^{*}=\frac{V_{m}\sigma }{\Delta G_{B}}$$

is obtained. At the nanoscale, the energy barrier between lithium and the lithiophilic metal decreases, which lowers the interfacial energy and consequently leads to the formation of finer lithium nuclei. These smaller nuclei possess higher surface energy, which enhances surface mobility and promotes atomic diffusion along grain boundaries. The growth of these finer lithium nuclei can be described by the strain rate of lithium due to a Coble creep behavior according to Equation (4)

(4) $$\frac{d\varepsilon }{dt}=K\frac{\delta_{s}D_{s}\Omega }{D^{3}k_{B}T}\sigma$$

in which *K*, δ_s_, D_s_, Ω, D, *k*_B_, T and σ represent the dimensionless constant, the nominal surface layer thickness, the surface diffusivity of lithium, the average grain diameter of lithium, the atomic volume of lithium, Boltzmann's constant, absolute temperature and the applied stress, respectively [46]. These finer lithium nuclei decrease the average grain diameter of lithium, thereby enhancing its creep behavior. The resulting creep‐driven redistribution enables more uniform lithium growth, thereby improving overall deposition stability [23, 47]. To provide theoretical support for this proposed creep mechanism, we performed machine‐learning nudged elastic band (ML‐NEB) calculations on 35 Li vacancy migration pathways across bulk and grain‐boundary structures. The results suggest that grain‐boundary diffusivity exceeds bulk diffusivity by several orders of magnitude, which is consistent with nanoscale Li deposits being located within the Coble creep regime on the Frost–Ashby deformation mechanism map (Note S3). However, during cycling, zinc particles may grow via Ostwald ripening, which could undermine the benefits of their initially small size by reducing the surface energy. As a result, this growth may ultimately lead to non‐uniform lithium deposition and reduced electrochemical stability (Figure 1c).

Here, we describe a systematic investigation aimed at developing an alternative to the silver‐carbon layer by selecting materials based on their intrinsic properties and optimizing their structural configuration. We synthesized a zinc oxide–carbon composite interlayer (ZnO@C) via the electron‐beam (e‐beam) irradiation method, which facilitated the formation of nanoscale ZnO particles on the carbon host. The nanoscale morphology is effectively preserved due to the strong chemical anchoring between ZnO and carbon, which suppresses Ostwald ripening‐induced agglomeration during cycling and improves overall interfacial stability. When the ZnO@C composite layer reacts with lithium metal, the conversion reaction of ZnO forms Li_2_O and a Li‐Zn alloy similar to Zn. The Li‐Zn alloy and Li_2_O exhibit both lithiophilic and MIEC characteristics, efficiently reducing local current density and guiding uniform lithium plating. Moreover, the lithiated ZnO nanoparticles, consisting of a Li‐Zn alloy and Li_2_O, are confined by strong bonding with carbon, which preserves their nanoscale morphology throughout cycling and promotes the Coble creep behavior of lithium beneath the ZnO@C interlayer without dendrite growth. Consequently, the ZnO@C composite layer exhibits a high CE of over 99.8% and stable capacity retention of 69.6% after 300 cycles.

## 2 Results and Discussion

**FIGURE 2:**
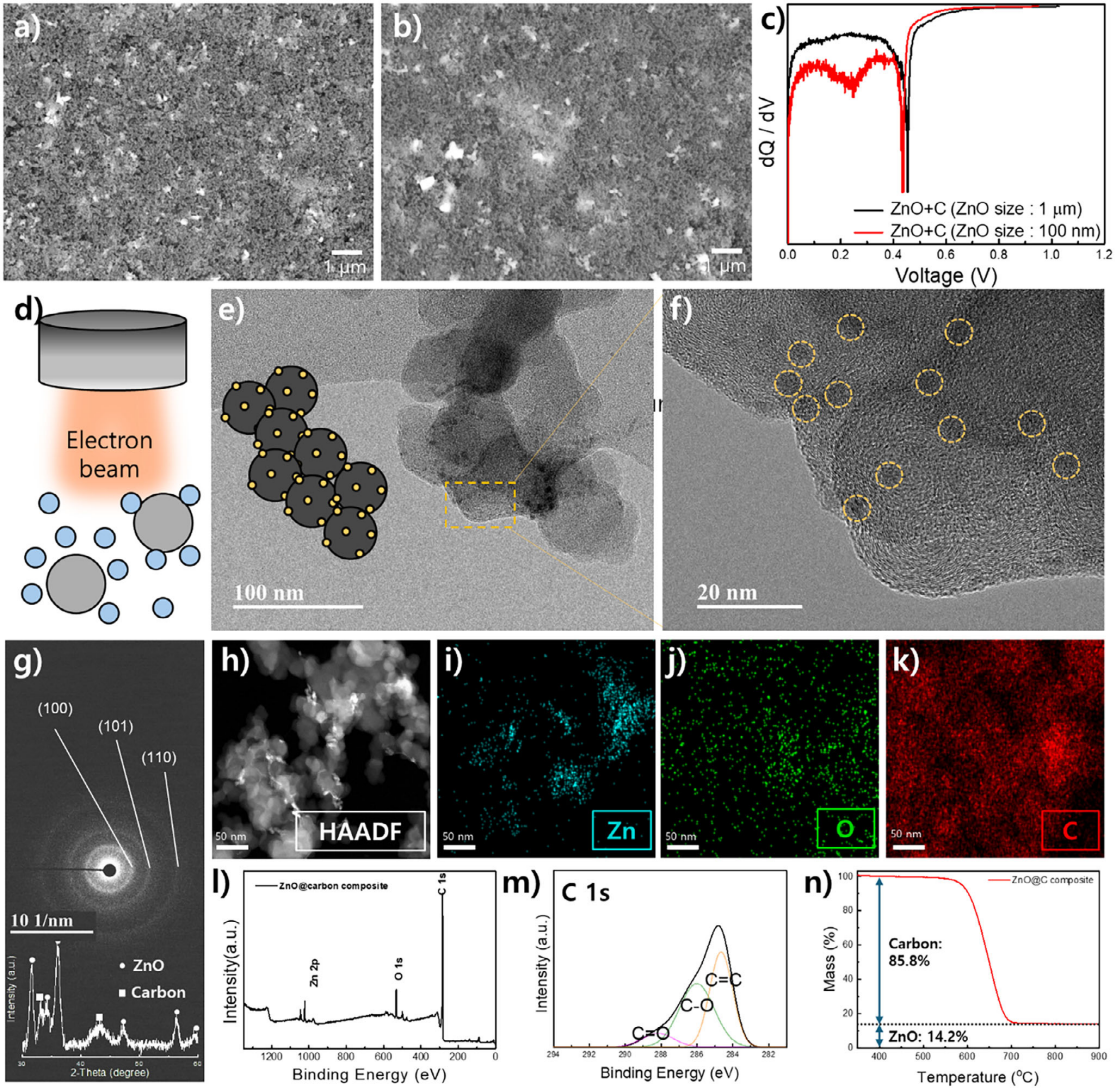
SEM images of ZnO+C (mixing) electrode using ZnO (a) 100 nm and (b) 1 µm particles. (c) Differential capacity plots (dQ/dV) during Li deposition on ZnO+C (mixing) electrode using ZnO 100 nm and 1 µm particles. (d) Schematic of ZnO@C composite synthesis using an electron beam. The (e) ZnO@C composite was characterized by (f) a high‐resolution TEM image. (g) Diffraction patterns of ZnO in ZnO@C. (h) High‐angle annular dark field and EDS maps of (i) zinc (blue), (j) oxygen (green), and (k) carbon (red). (l) XPS spectra of ZnO@C. m) XPS spectra of ZnO@C for C 1s. n) TGA results of ZnO@C.

Samples containing ZnO particles of approximately 100 nm and 1 µm were fabricated to examine how particle size influences their dispersion within the carbon matrix and the corresponding electrochemical behavior. Figures 2a and b show top‐view scanning electron microscopy (SEM) images of a control sample (ZnO+C) prepared by simply mixing carbon with 100 nm and 1 µm ZnO particles, respectively. The ZnO composite prepared with 100 nm ZnO particles exhibited a significantly more homogeneous dispersion of ZnO within the carbon matrix than the composite fabricated using 1 µm ZnO particles, which can be attributed to the higher specific surface area of the smaller particles that enhances interfacial contact with the carbon matrix and mitigates particle agglomeration during mixing. To examine the electrochemical behavior as a function of ZnO particle size, differential capacity (dQ/dV) plots were obtained and analyzed (Figure 2c). The alloying reaction with lithium and zinc, typically observed near 0.3 V [48], was not detected in the composite using 1 µm ZnO particles but was clearly visible in the composite prepared with 100 nm particles. Lithiation of carbon at approximately 0.4 V was observed in both composites, regardless of ZnO particle size. These results highlight the advantage of nanoscale ZnO in promoting alloying reactions with lithium. Guided by the above findings, ZnO was deliberately synthesized at the nanoscale in the design of the ZnO@C composite to enhance its electrochemical reactivity and promote homogeneous dispersion within the carbon matrix. To further ensure the long‐term stability of the nanoscale ZnO particles during cycling, the composite was designed such that ZnO and carbon are chemically bonded. E‐beam irradiation facilitated the formation of these strong chemical bonds and enabled the uniform dispersion of ZnO particles with controlled nanoscale dimensions throughout the composite (Figure 2d). To examine the structure of the synthesized ZnO@C composite, transmission electron microscopy (TEM) and high‐resolution TEM analyses were performed (Figure 2e,f). The as‐prepared ZnO@C composite consisted of a structure in which ZnO NPs are embedded within a carbon matrix. The carbon matrix had an approximate diameter of 70 nm, with ZnO NPs smaller than 5 nm uniformly embedded throughout. Electron diffraction pattern and X‐ray diffraction (XRD) were used to confirm the crystal structures of the ZnO in the ZnO@C composite. The electron diffraction pattern exhibited three main ring patterns corresponding to the (100), (101), and (110) planes of ZnO. The XRD spectrum in the inset of Figure 2g includes broad and high peak density for only the ZnO and amorphous carbon. The electron diffraction pattern and XRD results confirmed that the material consisted of moderately crystalline ZnO and amorphous carbon. The dispersion of ZnO and carbon particles in the ZnO@C was further examined using high‐angle annular dark field (HAADF) and energy‐dispersive spectroscopy (EDS) analyses. HAADF images of ZnO@C (Figure 2h) and EDS images of zinc (Figure 2i), oxygen (Figure 2j), and carbon (Figure 2k) revealed homogeneous dispersion of these elements within the ZnO@C composite. To further investigate the chemical characteristics of ZnO@C, X‐ray photoelectron spectroscopy (XPS) was used. The overall XPS results of ZnO@C shown in Figure 2l indicate the presence of only zinc, oxygen, and carbon within the ZnO@C structure. Figure 2m displays the C 1s XPS spectrum of ZnO@C, including three features associated with C═C, C─O, and C═O bonds at C1 ∼284.6 (284.5) eV, C2 ∼285.9 (285.8) eV, and C3 ∼288.4 (287.5) eV, respectively. The intensity of the C2 feature (C─O bond) in the C 1s XPS spectrum of ZnO@C composite is significantly greater than that of carbon black, suggesting modification of chemical states in the surface region due to the implantation of carbon. The XPS analysis confirmed chemical bonding between carbon and ZnO. Thermogravimetric analysis (TGA) revealed that the ZnO@C composite contained 14.2wt% ZnO NPs (Figure 2n).

**FIGURE 3:**
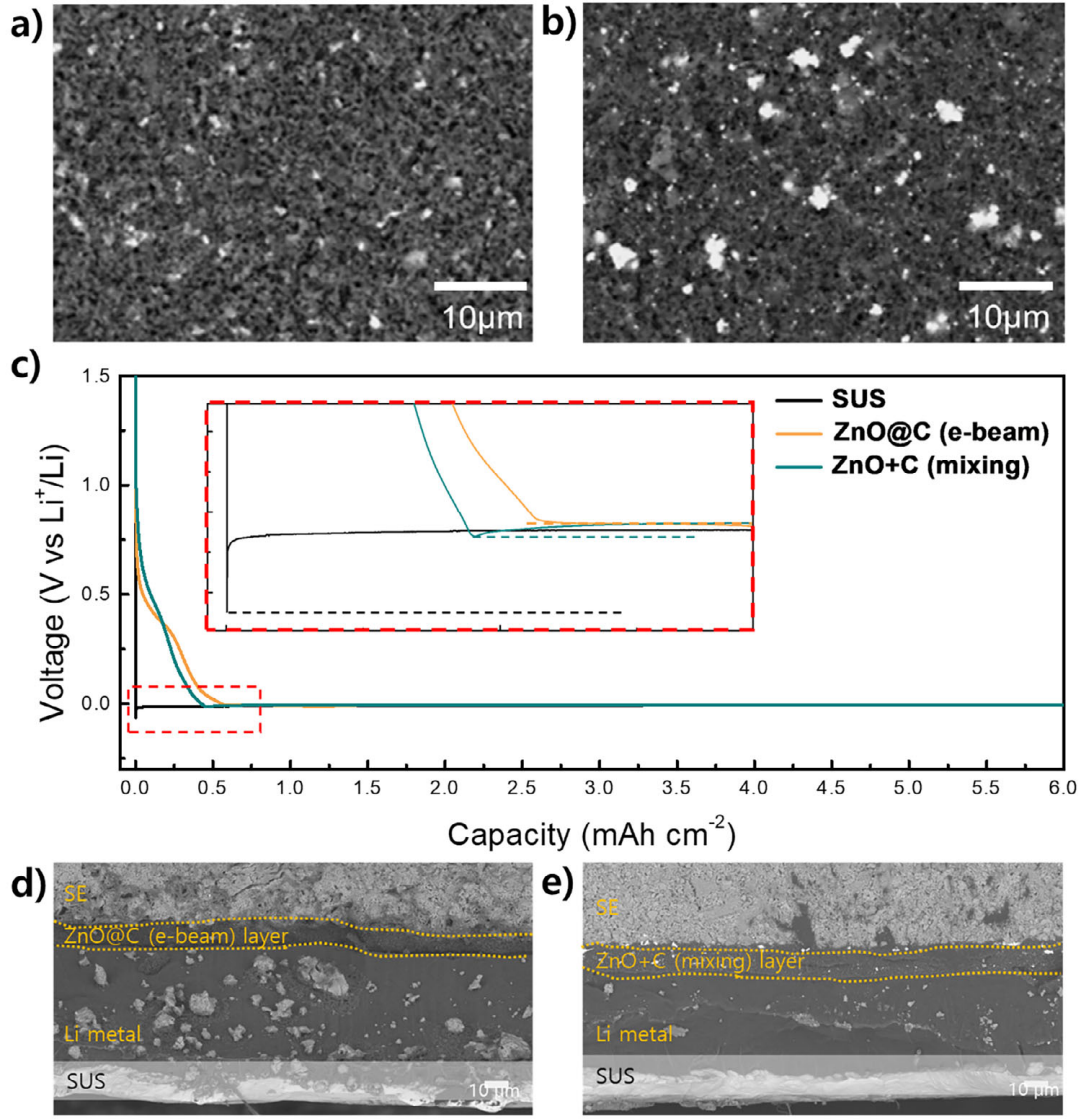
SEM images of (a) the ZnO@C (e‐beam) and (b) the ZnO+C (mixing) composite layer. (c) The voltage profile during lithium deposition on bare SUS, ZnO@C (e‐beam) and ZnO+C (mixing) electrodes at 0.3 mA cm^−2^. Cross‐sectional SEM images of (d) ZnO@C (e‐beam) and (e) ZnO+C (mixing) composite layers after lithium deposition.

Both ZnO@C and ZnO+C composites were prepared to compare the effect of e‐beam–assisted synthesis and evaluate their electrochemical behavior. The ZnO@C composite was synthesized using e‐beam irradiation, whereas the ZnO+C composite was prepared by simply mixing ZnO NPs with carbon black at the same ZnO content, without e‐beam treatment, resulting in no chemical bonding between the components. The resulting mixtures were processed into slurries, cast onto current collectors, and fabricated into electrodes for subsequent electrochemical testing. SEM top‐view images (Figure 3a) reveal that the ZnO@C composite layer achieved a significantly more uniform dispersion of ZnO NPs within the electrode structure compared with the ZnO+C composite layer. EDS mapping further confirmed that the ZnO embedded in the carbon matrix effectively suppressed ZnO NPs agglomeration during slurry preparation, leading to a homogeneous distribution of ZnO within the composite (Figure S3). Conversely, SEM images of the ZnO+C composite layer show considerable ZnO agglomeration, indicating more pronounced clustering than what was observed in the ZnO@C composite layer, and which could lead to uneven lithium deposition during cycling (Figure 3b). This agglomeration in the ZnO+C composite layer, characterized by clustered Zn particles, was also substantiated through EDS mapping (Figure S4). The improved uniformity in the ZnO@C composite layer can be attributed to the strong chemical bonds between ZnO and carbon. These bonds enable ZnO NPs to embed firmly within the carbon matrix, preventing agglomeration during slurry preparation and ensuring a stable, homogeneous dispersion throughout the composite. This chemical bonding allowed ZnO NPs to embed within a carbon matrix, stabilizing their distribution and reducing agglomeration during slurry preparation. In contrast, the ZnO+C composite layer, in which ZnO and carbon were physically mixed, experienced significant agglomeration due to the absence of chemical bonding.

The ZnO@C layer exhibited clear advantages in ensuring uniform and stable electrochemical performance. To evaluate the potential of ZnO NPs to stabilize lithium deposition, as well as the influence of their dispersion states on this effect, half‐cell experiments were conducted using lithium metal as the counter‐electrode. Lithium was deposited at a current density of 0.3 mA cm^−^^2^ until a total areal capacity of 6 mAh cm^−^^2^ was reached. Figure 3c shows the voltage profile of lithium metal deposition onto bare SUS, ZnO+C, and ZnO@C composite layers. When lithium metal was deposited onto bare SUS, a significant voltage drop was observed initially, followed by a flat voltage plateau. Additionally, to verify the lithium‐reactive nature of the ZnO@C composite layer, its electrochemical response during Li deposition was analyzed. The ZnO@C layer showed an initial reaction capacity of 0.6 mAh cm^−2^, which decreased to 0.2 mAh cm^−2^ in subsequent cycles (Figure S5). The nucleation overpotential of lithium metal corresponds to the difference between the lowest point of the voltage drop and the flat part of the plateau. This large nucleation overpotential led to non‐uniform lithium deposition, resulting in lithium that was neither dense nor uniform in shape on the SUS current collector (Figure S6). In contrast, the nucleation overpotential during lithium metal deposition on the ZnO@C and ZnO+C composite layers was nearly zero. After nucleation, the growth overpotential exhibited reduced polarization compared with the SUS electrode, indicating improved growth behavior for the ZnO@C and ZnO+C composite layers. Although the ZnO@C composite layer exhibited slightly lower resistance than the ZnO+C layer, this difference became negligible once lithium growth had occurred. This phenomenon suggests that zinc may have dissolved into the lithium metal prior to lithium deposition, potentially forming a solid solution with a crystal structure identical to that of lithium metal (Figure S7). This solid solution acts as a buffer layer for subsequent lithium deposition, effectively eliminating nucleation barriers. The ZnO@C composite layer, which is composed of ZnO NPs and carbon black, was introduced to the SUS to improve the stability of lithium deposition during cell operation. The solid‐solution reaction between ZnO and lithium metal reduced the nucleation overpotential, facilitating stable lithium deposition [13]. The slight difference in nucleation can be attributed to differences in the dispersion of ZnO NPs. When ZnO NPs aggregate, the surface area available for reaction with lithium is smaller than that of ZnO NPs uniformly distributed in the ZnO@C composite. This can lead to increased nucleation overpotential due to higher overvoltage during the formation of the lithium‐zinc alloy, resulting in non‐uniform lithium deposition (Figure S8). Figure 3d,e shows SEM images of lithium deposited on the ZnO@C and ZnO+C composite layers, respectively, both of which display uniform and dense morphologies. These observations visually confirm that the presence of ZnO lowers the nucleation overpotential and contributes to stable lithium deposition.

**FIGURE 4:**
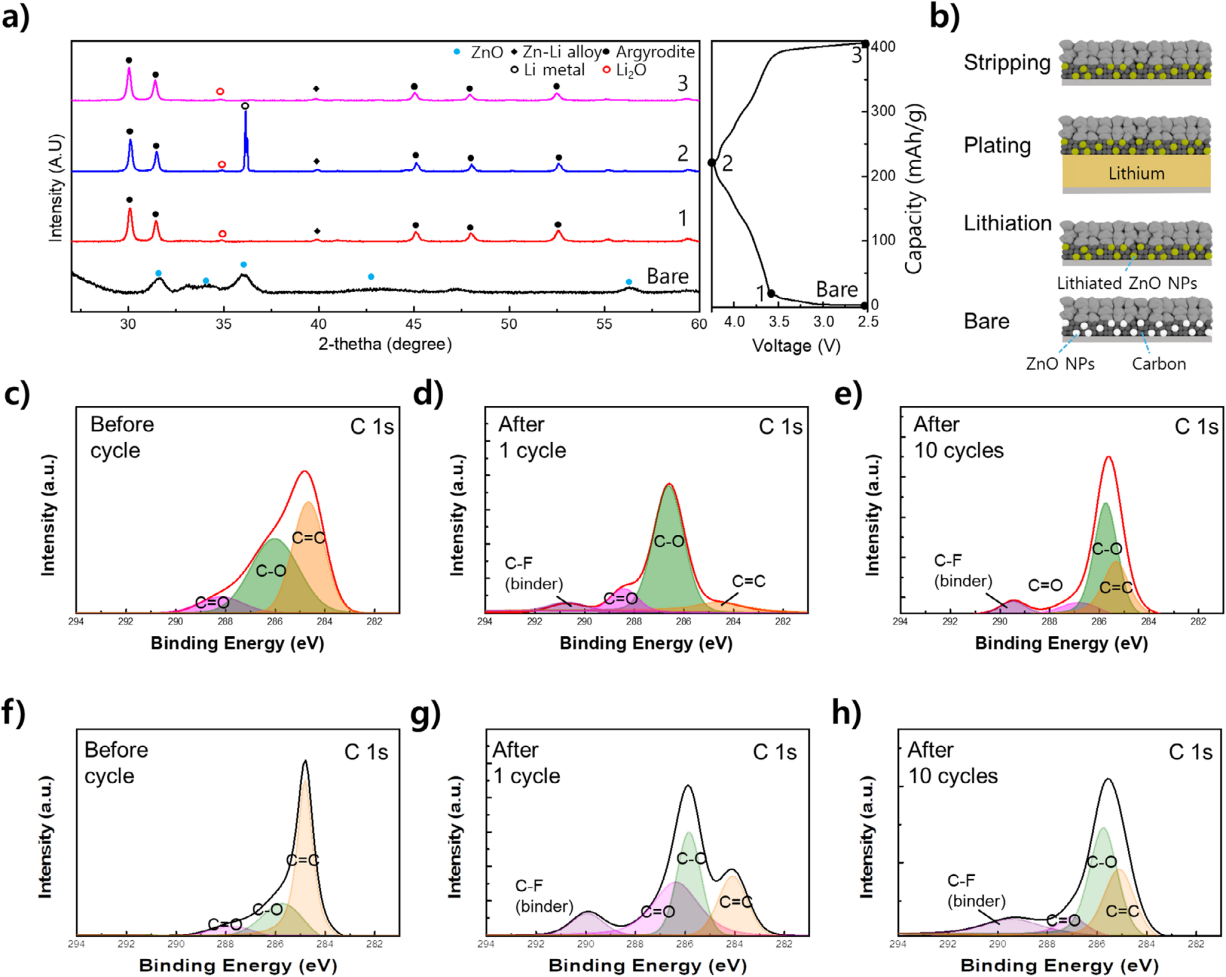
(a) X‐ray diffraction patterns of the AFSSB identify the phase transformation of the zinc‐lithium alloy during charging and discharging processes. (b) Schematic of lithium plating on the current collector with a ZnO@C (e‐beam) layer during charging processes. XPS spectra of ZnO@C (e‐beam) for (c) C 1s before cycle, (d) after 1cycle, and (e) 10 cycles. XPS spectra of ZnO+C (mixing) for (f) C 1s before cycle, (g) after 1cycle, and (h) 10 cycles.

To investigate the phase transformations occurring in the ZnO@C composite layer during lithium plating and stripping, XRD analysis was performed on a ZnO@C composite layer|solid‐electrolyte|cathode cell, with particular attention paid to the evolution of ZnO, Li_2_O, and zinc‐lithium alloy phases. (Figure 4a). Before charging (the bare state), ZnO peaks were observed at 33.1° and 47.0°. After the ZnO@C layer was fully lithiated (0.05 C, 22 mAh g^−^^1^) (1), new peaks appeared, indicating the formation of new intermetallic compounds such as a lithium‐zinc alloy and Li_2_O, while the existing ZnO peaks shifted or disappeared. Following 0.1 C charging (2), a new peak at 36° appeared, indicating the formation of lithium metal. During the subsequent discharge process (3), the peaks of the Li‐Zn alloy and Li_2_O were maintained, while the peak corresponding to lithium metal at 36° disappeared due to the migration of lithium metal. Figure 4b shows the schematic of lithium plating on the current collector with a ZnO@C layer during the charging process.

During charging, ZnO reacts with lithium according to the lithium‐zinc phase diagram, undergoing a solid solution reaction. We hypothesized that ZnO undergoes a conversion reaction with lithium to form a Li‐Zn alloy and Li_2_O, both of which are uniformly embedded within the carbon matrix at the nanoscale. These products subsequently interact with additional lithium metal to form a solid solution, thereby facilitating uniform lithium plating. The Li‐Zn alloy and Li_2_O formed during this process possess high ionic conductivity, which helps stabilize lithium deposition. Due to the chemical bonding between ZnO and carbon, the ZnO NPs dispersed uniformly within the carbon matrix, allowing the subsequent conversion reaction to occur in a spatially controlled manner. As a result, the lithiated ZnO NPs, consisting of a Li‐Zn alloy and Li_2_O, were also uniformly distributed at the nanoscale, further enhancing the homogeneity and stability of lithium deposition. Subsequently, lithium was deposited, and during the discharge process, the lithium metal layer completely disappeared (Figure S9). XPS analysis was performed on the ZnO@C composite before and after cycling to examine changes in the chemical states related to lithium deposition. Figure 4c–h presents the XPS spectra of the C 1s states and their fitted bonding states for the ZnO@C layer and ZnO+C composite layer before cycling, after 1 and 10 cycles, respectively. The C 1s XPS analysis, based on the survey scan of ZnO@C and ZnO+C composites, includes a characteristic peak at approximately 284.5 eV. Additionally, the line shape of the main feature of the C 1s state in the XPS spectrum broadened after cycling, indicating that carbon‐related chemical states were induced in the surface region by the implantation of carbon atoms into ZnO@C. The fitted C 1s XPS spectrum of ZnO@C included three peaks corresponding to C═C, C─O, and C═O bonds at C1 ∼284.6 (284.5) eV, C2 ∼285.9 (285.8) eV, and C3 ∼288.4 (287.5) eV, respectively. The C2 peak (C─O bond) intensity in the C 1s XPS spectrum of the ZnO@C composite was substantially higher than that of the ZnO+C composite, indicating a modification of surface chemical states induced by the ZnO embedded onto the carbon matrix. This enhanced C2 intensity persisted in ZnO@C even after 1 and 10 cycles, implying the formation and retention of stable chemical bonds between lithiated ZnO NPs and carbon throughout prolonged operation. In the XPS spectra of the ZnO@C and ZnO+C composite, the main feature of the O 1s state (Figure S10) shifted to a higher binding energy in the ZnO@C composite. The intensity of the O2 feature in the O 1s XPS spectrum increased, a change that is associated with the C2 feature in the C 1s XPS spectrum of ZnO@C, supporting the proposal that carbon implantation generates more C─O‐related bonds in the surface region. Consequently, the formation of C─O bonds is favored, and the number of unpaired and/or dangling bonds at oxygen sites is reduced in the ZnO@C composite. The XPS spectra of the C 1s states and their fitted bonding states after a single cycle and 10 cycles are also presented.

**FIGURE 5:**
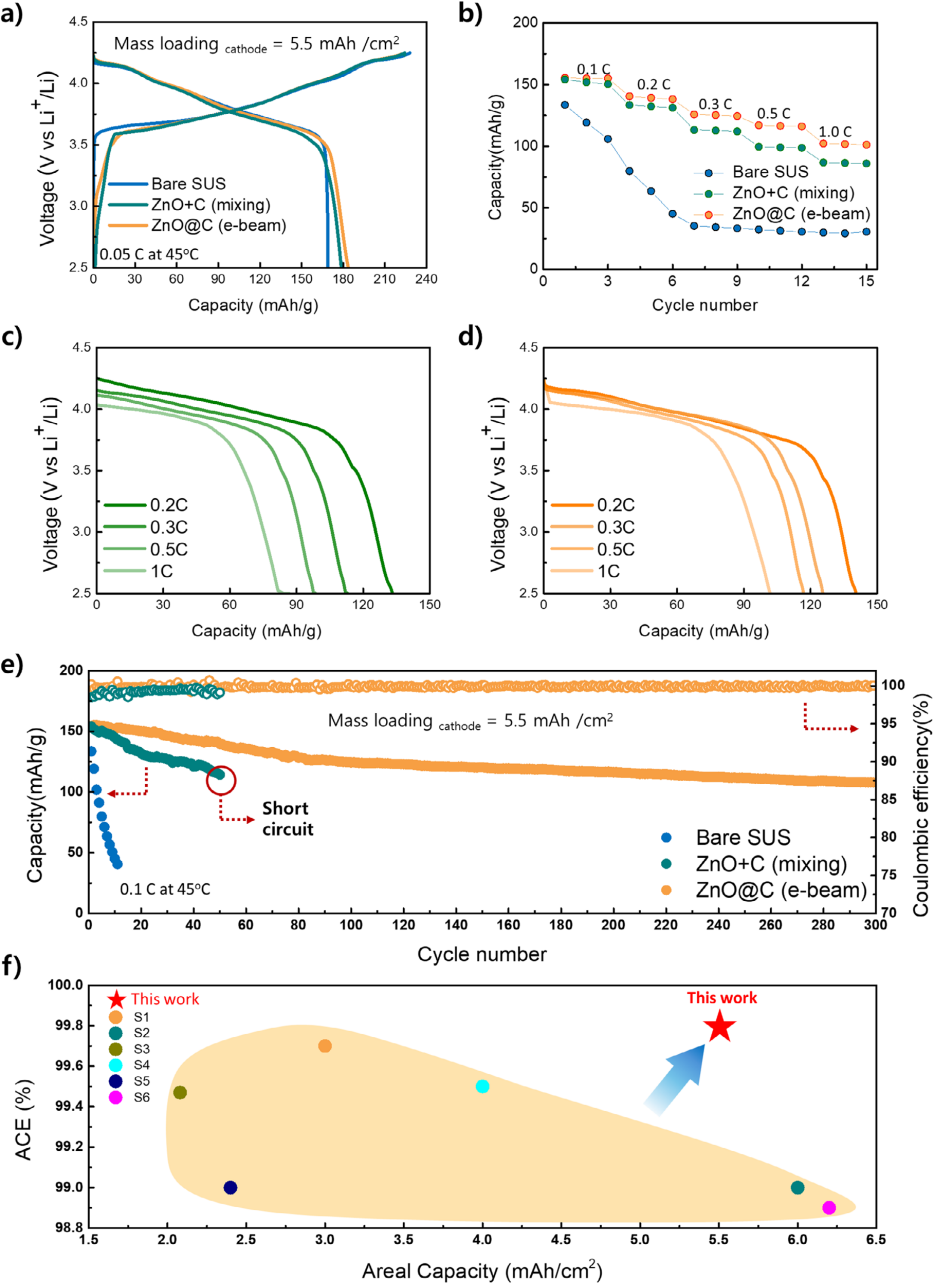
Electrochemical properties of AFSSBs using an NMC9055‐SE composite cathode and bare SUS, ZnO+C (mixing), and ZnO@C (e‐beam) composite layer. (a) Initial voltage profiles for the AFSSBs at 0.05 C. (b) Discharge capacities at current rates of 0.1 C, 0.2 C, 0.3 C, 0.5 C, and 1 C. Voltage profiles of (c) ZnO+C (mixing) and (d) ZnO@C (e‐beam) composite layers were plotted with specific capacity. (e) Cycle performances for 300 cycles at 0.1 C (1 C = 5.5 mA cm^−2^). (f) Comparison of areal capacity and average Coulombic Efficiency (ACE) performance in this work with other works of literature.

The electrochemical performance of the ZnO@C and ZnO+C composite layers was comparatively evaluated using full cells assembled with SE and an NMC cathode (5.5 mAh cm^−2^ areal capacity). Figure 5a shows the initial charge‐discharge voltage profiles at 0.05 C. The full cells employing bare SUS, ZnO+C, and the ZnO@C composite layer delivered discharge capacities of 168.87 mAh g^−1^, 178.47 mAh g^−1^, and 183.62 mAh g^−1^, with initial respective Coulombic efficiencies of 74.0%, 75.1%, and 81.9%. The higher initial capacity and Coulombic efficiency of the ZnO@C composite layer can be attributed to the homogeneous dispersion of nanosized ZnO within the carbon matrix, which facilitated rapid charge transfer and more stable lithium plating and stripping behavior. The rate capabilities of the AFSSBs were evaluated at various current rates (Figure 5b). By varying the discharge rate from 0.1 C (0.55 mA cm^−2^) to 1.0 C (5.5 mA cm^−2^), the performance was examined between 2.5 V and 4.25 V at a fixed charge rate of 0.1 C (0.55 mA cm^−2^) in constant current (CC)–constant voltage (CV) mode. The full cell employing the ZnO@C composite layer exhibited a significantly improved rate capability compared to the full cell employing the bare SUS and ZnO+C composite layer at all current rates. Specifically, the cell using the ZnO@C composite layer delivered average charge capacities of 155.09 mAh g^−^^1^, 139.25 mAh g^−^^1^, 125.15 mAh g^−^^1^, 116.50 mAh g^−^^1^, and 101.75 mAh g^−^^1^ at 0.1 C, 0.2 C, 0.3 C, 0.5 C, and 1 C at 45°C, respectively. In contrast, the cells with the bare SUS and ZnO+C composite layer delivered average charge capacities of 106.38 mAh g^−1^, 36.51 mAh g^−1^, 12.39 mAh g^−1^, 10.60 mAh g^−1^, and 10.35 mAh g^−1^ and 151.94 mAh g^−1^, 132.28 mAh g^−1^, 112.69 mAh g^−1^, 98.97 mAh g^−1^, and 86.41 mAh g^−1^ at the same current rates at 45°C, respectively. Figure 5c,d displays the voltage profiles of the full cells employing ZnO+C and ZnO@C composite layers. The ZnO+C composite layer exhibits a gradual decrease in discharge capacities with increasing c‐rate, along with increased polarization, indicating sluggish reaction kinetics at a high current density. In contrast, the ZnO@C composite layer delivered significantly more stable voltage profiles at all c‐rates, maintaining higher discharge capacities even under high current density. The cycle performance of the cells at a current density of 0.1 C (Figure 5e) was also monitored. The cell using a ZnO@C composite layer had a capacity retention of 69.6% after 300 cycles at 45°C at a 0.1 C rate. Notably, the AFSSB also maintained a CE of greater than 99.8% after 100 cycles, which can be attributed to stable lithium metal deposition. However, the cell with the bare SUS experienced a sharp capacity drop after just 10 cycles, while the ZnO+C composite layer exhibited a similar sharp capacity drop after 50 cycles. This sharp decline in capacity can be attributed to the agglomeration of ZnO NPs during cycling, which increased the energy barrier and prevented effective lithium creep behavior, leading to rapid capacity loss and potential short‐circuiting. Suppressing this Ostwald ripening effect can help the AFSSB maintain superior cycle performance and CE, indicating that the AFSSB with a ZnO@C composite layer can achieve superior cycling performance compared with one using a bare current collector as the anode. The capacity and cycle performance of the ZnO@C composite layer are similar to those of the Ag+C (mixing) layer (Figure S11), and both exhibit better electrochemical performance than the Li metal anode. The energy density of the resulting AFSSBs employing the ZnO@C composite layer was calculated using the parameters listed in Table S1, including the thickness of the ZnO@C composite layer. The ZnO@C composite layer exhibited an improved volumetric energy density of 925 Wh L^−1^ compared with 752 Wh L^−1^ for the Li metal anode. These results indicate that the use of the lower‐cost ZnO, including the e‐beam irradiation process, can achieve comparable performance and is therefore advantageous for practical commercialization. (Table S2). Figure 5f summarizes the performance of AFSSBs in achieving the areal capacity and average Coulombic Efficiency (ACE). The full cells employing the ZnO@C composite layer show higher areal capacity and average Coulombic Efficiency (ACE) performance than those reported in previous studies.

**FIGURE 6:**
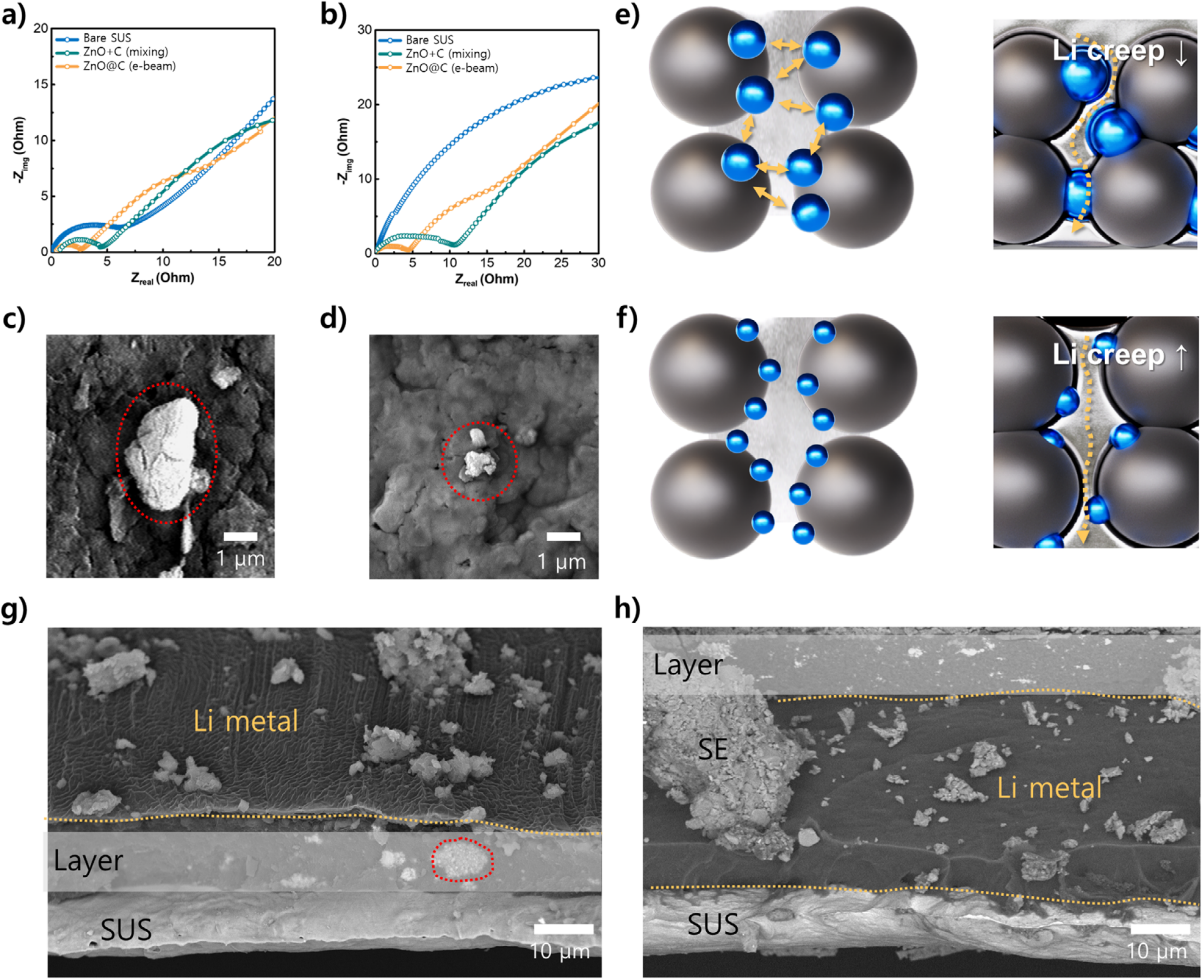
EIS spectra of AFSSBs using a bare SUS, ZnO+C (mixing), and ZnO@C (e‐beam) composite layer after (a) 1 and (b) 10 cycles. SEM image of (c) ZnO+C (mixing) and (d) ZnO@C (e‐beam) composite layer after 10 cycles of stripping. Schematic of particle agglomeration behavior induced by Li creep during cycling in (e) ZnO+C (mixing) and (f) ZnO@C (e‐beam) composite layers. Cross‐sectional SEM image of plated lithium after 10 cycles on the (g) ZnO+C (mixing) and h) ZnO@C (e‐beam) layer after 0.3 mA cm^−2^ charging at 6 mAh cm^−2^.

Electrochemical post‐analysis was conducted through electrochemical impedance spectroscopy (EIS) analysis (Figure 6a,b). After the deposition of lithium metal, charge transfer dominated the low‐frequency region due to the non‐blocking nature of lithium metal, which appeared as a semi‐circular feature at low frequencies. Based on the diameter of the semi‐circle, the interfacial resistance between the deposited lithium on bare SUS and the solid electrolyte surface was found to be 8–10 Ω cm^2^, while the interfacial resistance of lithium deposited on the ZnO+C composite layer against the solid electrolyte was 4–5 Ω cm^2^. In contrast, the interfacial resistance of lithium deposited on the ZnO@C composite layer against the solid electrolyte was 2–3 Ω cm^2^. This confirms that the ZnO@C composite layer facilitates uniform lithium metal deposition during charging. During cell operation, the interfacial resistance of the bare SUS increased significantly due to the accumulation of voids and dendrites from lithium metal deposition, leading to a short circuit. The interfacial resistance of the ZnO+C composite layer increased significantly to 10–11 Ω cm^2^. However, the interfacial resistance of the ZnO@C composite layer remained at a similar level even after cycling, increasing only slightly to 3–4 Ω cm^2^ compared with the initial cycle. This indicates that the ZnO@C composite layer helps form uniform and dendrite‐free lithium metal plating. The difference in interfacial resistance can be attributed to the increased energy barrier caused by the agglomeration of lithiated ZnO NPs through Ostwald ripening during cycling. SEM images were used to examine lithiated ZnO NPs within the ZnO+C (Figure 6c) and ZnO@C (Figure 6d) composite layers. After 10 cycles, the lithiated ZnO NPs in the ZnO@C composite layer remained approximately 200 nm in size due to chemical bonding with carbon, whereas those in the ZnO+C composite layer grew to approximately 3 µm. This increase in particle size affected the creep behavior, leading to deteriorated creep behavior after 10 cycles. Figure 6e,f presents schematic illustrations showing that the enlarged lithiated ZnO NPs generated by Ostwald ripening increase the tortuosity of the pathways for creep deformation in the composite layer, ultimately hindering the creep progression of lithium. Cross‐sectional SEM images were taken after depositing lithium for 10 cycles to observe the lithium deposition behavior in ZnO+C (Figure 6g) and ZnO@C composite layers (Figure 6h). In the ZnO+C composite layer, the cross‐sectional SEM results revealed that lithium deposition occurred between the ZnO+C composite layer and the solid electrolyte, indicating no creep behavior. Deposition of lithium between the layer and the solid electrolyte can lead to side reactions with the solid electrolyte, increasing the interfacial resistance. In contrast, the ZnO@C composite layer continuously exhibited creep behavior, allowing lithium to be deposited stably between the layer and SUS, thereby maintaining low interfacial resistance. The suppression of Ostwald ripening facilitated continuous creep behavior, enabling higher electrochemical performance.

## 3 Conclusion

We demonstrated that zinc reacts with lithium metal through a solid solution similar to silver in anode‐free all‐solid‐state lithium metal batteries with a high energy density. The formation of a dense lithium metal layer, which could be moved repeatedly between the zinc oxide–carbon composite interlayer (ZnO@C) and the current collector, was demonstrated. During charging, zinc was alloyed with lithium in the solid solution. These findings indicate that our design produces a stable anode capable of preventing uneven lithium metal deposition. The cells exhibited a high energy density and long‐term cycling compared with silver NPs. This work creates the potential for developing batteries with a high energy density and low cost.

## 4 Experimental Section and Methods

### 4.1 Synthesis of ZnO@C Composite Powder by Electron‐Beam (e‐Beam) Irradiation Method

The ZnO@C composite powder was prepared via electron‐beam irradiation under different absorbed doses. First, 3 g of PVP (3 × 10^−4^ mol) was dissolved in 100 mL of ethylene glycol. Subsequently, 5 g of Zn(CH_3_COO)_2_·2H_2_O (0.02 mol) and 15 mL of isopropyl alcohol were added to the PVP solution. After stirring the mixture for 30 min, nitrogen gas was purged through the solution for an additional 30 min to eliminate dissolved oxygen. The resulting solution was then exposed to electron‐beam irradiation at various absorbed doses. After irradiation, the ZnO@C composite powder was separated by centrifugation, washed three times with ethanol, and dried under vacuum for 3 h to obtain the final product. In this work, electron‐beam irradiation was carried out using a KORENS RTX electron‐beam accelerator, which operated at an electron energy of 1 MeV and a beam current of 1 mA under atmospheric conditions.

### 4.2 Preparation of the ZnO@C Composite Layer

A ZnO@C composite powder (KORENS RTX) was prepared as the composite layer. ZnO@C powder was mixed in N‐Methyl‐2‐pyrrolidone (DAE JUNG), containing 7 wt% of polyvinylidene fluoride (Kureha). N‐Methyl‐2‐pyrrolidone was added slowly to the mixture under constant stirring using a mixer (ARE‐300, Thinky Corporation, Japan) to prepare the slurry. The slurry was then coated onto a SUS foil 10 µm thick using a screen printer and dried in the air at 60°C for 2 h. The obtained electrode was further dried under vacuum at 120°C for 6 h. The amount of ZnO@C composite layer used in cell preparation was 1 mg/cm^2^.

### 4.3 Materials Characterization

The ZnO@C composite layer and all‐solid‐state batteries were physically cut and sampled, and the cross‐section of the samples was observed by SEM (JSM‐7600F, JEOL, Japan). XRD analysis (D8 ADVANCE, Bruker, USA) was used to confirm the material phase. The ratio of zinc content in the ZnO@C composite layer was confirmed through TGA (STA 449 F3, Netzsch, Germany). The TGA analysis conditions included a temperature increase rate of 5°C/min, followed by carbon burnout sustained at 1300°C.

### 4.4 Electrochemical Evaluation

AFSSBs were fabricated in a dry, argon‐filled glovebox. For fabrication of cathode composite electrodes, NMC9055 (Ni_0.90_Mn_0.05_Co_0.05_)O_2_, an argyrodite‐type Li_6_PS_5_Cl solid electrolyte (99 wt%, 3 µm, Mitsui Mining & Smelting Co., Ltd), and carbon nanofibers (Sigma‐Aldrich) were mixed at a weight ratio of 65:35:5. The synthesized solid electrolyte showed a conductivity of 1.92 × 10^−3^ S cm^−1^ (Figure S12), and the argyrodite phase was confirmed by XRD measurements (Figure S13). The ZnO@C composite layer was used as the anode. First, the solid electrolyte was placed in a polycarbonate tube and subjected to 50 MPa of pressure. Next, the cathode composite electrode was put on the side of the SE layer and pressed at 300 MPa. Finally, the ZnO@C composite layer was attached to the side of SE by pressing at 50 MPa (Figure S14a). Electrochemical performances of the samples in AFSSBs were investigated by galvanostatic testing using a TOSCAT 3000 battery tester (TOSCAT 3000, Toyo Systems, Japan). The cutoff voltage was set to between 2.5 V and 4.25 V versus lithium. To measure rate capability, the current density was changed from 0.1 C to 1 C at 45°C. Half cells were fabricated in a dry, argon‐filled glovebox. The SE layer was formed at a pressure of 300 MPa alone in a polycarbonate tube, and the ZnO@C composite layer and lithium metal were then attached to each side and subjected to 40 MPa of pressure (Figure S14b). The electrochemical performance was investigated using a TOSCAT 3000 battery tester (TOSCAT 3000, Toyo Systems, Japan) with galvanostatic conditions at 45°C. The impedance of the AFSSBs was measured using EIS and a potentiostat/galvanostatic (Metrohm AG, Autolab, The Netherlands) over a frequency range of 1 MHz to 0.01 Hz at an amplitude of 10 mV.

### 4.5 Computational Methods

The DFT calculations were carried out with the Quantum Espresso software package [49]. The Generalized gradient approximation (GGA) in the Perdew–Burke–Ernzerhof (PBE) parametrization and the projected augmented wave (PAW) method were used to describe the electron exchange‐correlation and electron‐ion interaction. For the adsorption calculation, we considered the van der Waals interactions using the DFT‐D3(BJ) method. The DFT was conducted with a Li [111] slab model on a 3 × 3 × 4 Li superlattice.

The activation energy barriers for lithiation were determined using the CI‐NEB method, which is a robust technique for identifying minimum energy paths (MEPs) and transition states. These calculations were performed using the Quantum Espresso suite, specifically employing the Plane‐Wave Self‐Consistent Field (PWSCF) solver to ensure uniform computational accuracy throughout the study. (Table S3) Four intermediate images were interpolated between the initial and final states to accurately map the MEPs. A string method, combined with a ‘quick‐min’ optimization approach, was used to converge the images efficiently toward the MEP. The spring constants were carefully selected, ranging from 0.2 to 0.3, ensuring an even distribution of images along the energy path. The optimization process utilized a step size of 2.0 atomic units, which allowed for a detailed examination of the energy landscape. Each CI‐NEB calculation was initialized independently, with a single step performed per iteration to closely monitor convergence and make necessary parameter adjustments.

### 4.6 Machine‐Learning NEB and Coble Creep Calculations

Grain‐boundary (GB) Li‐ion migration barriers were calculated using the nudged elastic band (NEB) method accelerated by the MACE‐MP‐0 universal machine‐learning interatomic potential (medium variant, float64 precision). Three coincidence‐site lattice (CSL) bicrystal models (sigma3 and sigma9) were constructed with the aimsgb package and relaxed to a force convergence of 0.01 eV/A. A total of 35 symmetry‐inequivalent vacancy migration pathways were enumerated across the three GB types. Climbing‐image NEB (CI‐NEB) calculations were performed with 7 intermediate images initialized via the image‐dependent pair potential (IDPP) method, using the FIRE optimizer (fmax = 0.01 eV/A, maximum 150 steps).

Because MACE‐MP‐0 systematically underestimates barrier heights relative to experiment, a barrier ratio scaling approach was adopted. The scaling factor alpha = E_a_ (exp) / E_bulk_ (ML) = 0.557/0.0564 = 9.88 was determined from the ratio of the experimental bulk Li self‐diffusion activation energy (E_a_ = 0.557 eV) to the ML‐computed bulk vacancy migration barrier. Scaled GB barriers were then obtained as E_GB_ (scaled) = alpha x E_GB_ (ML). This approach preserves the physically meaningful dimensionless barrier ratio E_GB/_E_bulk_, which is insensitive to the systematic bias of the ML potential.

Temperature‐dependent diffusion coefficients were calculated via the Arrhenius relation D = D_0_·$e^{-E_{a}/k_{B}T}$ with D_0_ = 4.4 × 10^−5^ m^2^/s. Coble creep rates were computed using the classical equation with K = 148, Omega = 2.18 × 10^−29^ m^3^, and delta = 5 × 10^−10^ m. A Frost & Ashby deformation mechanism map was constructed to identify the dominant creep mechanism at the AFSSB operating conditions (T = 318 K, sigma = 10 MPa).

Li adsorption energies on ZnO conversion product surfaces (Li_2_O, LiZn) were calculated using MACE‐MP‐0 with slab models. The Li migration barrier through Li_2_O (0.31 eV) was adopted from literature density functional theory calculations to assess the ion‐transport capability of the conversion products. Full computational details and results are provided in Note S3.

## Funding

This work was supported by the Ministry of Trade, Industry and Energy of the Republic of Korea (No. 20012328, Development of lithium friendly 100 nm scale metal composite anode system for all solid state batteries using electron beam) and this work was also supported by the Nano & Materials Technology Development Program through the National Research Foundation of Korea (NRF) funded by the Ministry of Science and ICT [RS‐2024‐00449682 (50%)]. This work was also supported by a Korea Institute for Advancement of Technology (KIAT) grant funded by the Korea Government (MOTIE) (RS‐2024‐00417730, HRD Program for Industrial Innovation) and “Regional Innovation System & Education (RISE)” through the Seoul RISE Center, funded by the Ministry of Education (MOE) and the Seoul Metropolitan Government. (2026‐RISE‐01‐027‐01). Additionally, this work was supported by the research fund of Hanyang University and KBSI (HY‐202500000003967).

## Conflicts of Interest

The authors declare no conflicts of interest.

## Supporting information




**Supporting File**: advs75982‐sup‐0001‐SuppMat.docx.

## Data Availability

The data that support the findings of this study are available in the supplementary material of this article.
