# Supplementary material for: Carbon Interlayer with Uniformly Anchored ZnO Nanoparticles: Surface‐Energy‐Driven Coble Creep for Practical Anode‐Free Solid‐State Batteries
Source: Adv Sci (Weinh). 2026 Jun 3;13(48):e00057. doi: 10.1002/advs.202600057 (PMC13337064; doi:10.1002/advs.202600057)
Supplement: Supplementary file 1 [file ADVS-13-e00057-s001.docx]

Supporting Information

**Surface-energy-driven lithium creep behavior beneath the interlayer for practical anode-free solid-state batteries**

*Joonhyeok Park, Jeongheon Kim, Seungwoo Lee, Jaeik Kim, Il Woo Ock, Seunggun Choi, Jooheon Sun, Gunwoo Cha, Seungmin Han, Hyungjun Lee, Jun Lim, Hyun-Suk Kang, Jiung Cho, Jong Sung Jin, Seho Sun*, Ungyu Paik* and Taeseup Song**

J. Park, J. Kim, S. Lee, J. Kim, I. Ock, S. Choi, J. Sun, G. Cha, S. Han, H. Lee, U. Paik, T. Song

Department of Energy Engineering, Hanyang University, Seoul 04763, Republic of Korea

E-mail: [tssong@hanyang.ac.kr](mailto:tssong@hanyang.ac.kr), [upaik@hanyang.ac.kr](mailto:upaik@hanyang.ac.kr)

J. Lim, T. Song
Department of Battery Engineering, Hanyang University, Seoul 04763, Republic of Korea

E-mail: [tssong@hanyang.ac.kr](mailto:tssong@hanyang.ac.kr)

H. Kang

Korens RTX R&D Center, Gyeryong 32842, Republic of Korea

J. Cho

Department of Materials Science and Engineering, Hongik University, Sejong 30130, Republic of Korea

J. Jin

Busan Center, Korea Basic Science Institute, Busan 46742, Republic of Korea

S. Sun

School of Chemical Engineering, Yeungnam University, Gyeongsan 38541, Republic of Korea

E-mail: [seho.sun@yu.ac.kr](mailto:seho.sun@yu.ac.kr)

**Supplementary Notes**

**Supplementary Note 1: Lithiophilic metal candidates**

600 nm thick layers of Ag, Sn, In, and Zn were deposited via e-beam evaporation, followed by lithium deposition to evaluate suitable lithiophilic metal candidates. In and Zn exhibited reactions comparable to Ag during lithium deposition (**Figure N1**). Half-cell tests were conducted by depositing 6 mAh cm⁻² of lithium onto each candidate metal to examine the nucleation overpotential (**Figure N2**). Ag showed an almost negligible nucleation overpotential, while In and Zn also displayed values close to zero. In contrast, Sn exhibited a slightly higher nucleation overpotential. Differential capacity (dQ/dV) analyses were further performed to investigate the alloying reaction potentials between lithium and the candidate metals (**Figure N3**). Ag exhibited alloying reactions with lithium below 0.1 V, whereas Zn showed alloying peaks both below 0.1 V and at ~0.2 V. In, however, revealed alloying peaks around 0.6 V, while Sn exhibited peaks above 0.4 V. Full-cell tests using NMC as the cathode confirmed that both In and Zn achieved discharge capacities and efficiencies comparable to Ag (**Figure N4**). Nevertheless, In reacted at higher potentials relative to Ag, implying insufficient robustness as a buffer layer for lithium deposition.[7] Furthermore, considering its higher cost, Zn was identified as the most practical candidate.

**Figure N1.** Scheme and cross-sectional SEM images after deposited lithium on the a) Ag, b) Sn, c) In and d) Zn layer after 0.3 mA cm^-2^ charging at 6 mAh cm^¬2^

**Figure N2.** Voltage profile during Li deposition on a) Ag, b) Sn, c) In and d) Zn layer at 0.3 mA cm^-2^ charging at 6 mAh cm^¬2^

**Figure N3.** Differential capacity plots (dQ/dV) during Li deposition on a) Ag, b) Sn, c) In and d) Zn layer.

**Figure N4.** Initial voltage profiles of Ag, Sn, In and Zn layer for the AFSSBs at 0.05 C.

**Supplementary Note 2: Ag/Zn lithiation barrier energy DFT calculation**

The Li deposition on the upper interlayer leads to increased side reactions with the solid electrolyte, resulting in poor lifespan characteristics and suboptimal battery performance. Conversely, incorporating lithiophilic particles into the carbon layer transforms the deposition dynamics. These particles act as nucleation sites for Li deposition, promoting a more uniform Li distribution and reducing the likelihood of side reactions with the solid electrolyte. Directed deposition facilitated by lithiophilic particles significantly enhances creep behavior, allowing Li to migrate along the particles and redistribute mechanical stress more effectively. Among the lithiophilic metal candidates, Ag and Zn are prominent due to their lower surface energy at the Li-metal and Li-M alloy junctions compared to other metals. As shown in **Figure N5**, Zn has been reported to underperform in anode-free cells due to its higher sub-lattice diffusion barrier energy compared to Ag (Ag: 4.807 eV, Zn: 6.458 eV). To use Zn as a substitute for Ag, both microscopic and macroscopic controls are necessary.

**Figure N5.** DFT calculation of energy barrier, structural evolution and bonding preference for atoms in Ag and Zn with Li during lithiation

**Supplementary Note 3: Computational Evidence for Coble Creep in Nanocrystalline Lithium**

**SN3.1 Methodology Overview**

To quantitatively evaluate the Coble creep hypothesis, we combined machine-learning nudged elastic band (ML-NEB) calculations with experimental scaling to determine grain-boundary (GB) diffusion parameters in nanocrystalline lithium. The workflow consists of three stages: (i) ML-NEB screening of vacancy migration barriers across representative GB types (Sigma3, Sigma9), (ii) experimental scaling to convert ML barriers to physically accurate activation energies, and (iii) analytical Coble creep modeling using the scaled diffusion coefficients.

**SN3.2 ML Interatomic Potential**

All NEB calculations employed the MACE-MP-0 foundation model (medium variant), a universal machine-learning interatomic potential pre-trained on the Materials Project database covering 89 elements.[8] Calculations were performed with float64 precision on NVIDIA RTX 5090 GPUs. While MACE-MP-0 systematically underestimates absolute barrier heights (by approximately one order of magnitude relative to experiment), it preserves the relative ordering of barrier heights across different GB types, making the dimensionless barrier ratio E_GB_/E_bulk_ a reliable descriptor.

**SN3.3 Grain Boundary Construction**

Three coincidence-site lattice (CSL) grain boundaries were constructed using the aimsgb package:

| GB Type | Sigma | Rotation Axis | Tilt Angle | Supercell Atoms | GB Area (A^2^) |
| --- | --- | --- | --- | --- | --- |
| Sigma3 | 3 | [1 1 0] | 70.5^o^ | ~48 | — |
| Sigma9 | 9 | [1 1 0] | 38.9^o^ | ~144 | — |

Each bicrystal supercell was relaxed with MACE-MP-0 to a force convergence of 0.01 eV/A. Vacuum layers of 15 A were applied perpendicular to the GB plane to avoid periodic image interactions. A single vacancy was introduced at each symmetry-inequivalent site near the GB core, and all nearest-neighbor and next-nearest-neighbor vacancy hop paths were enumerated, yielding a total of 35 candidate migration pathways.

**SN3.4 NEB Calculations**

Climbing-image NEB (CI-NEB) calculations were performed using the ASE NEB module with 7 intermediate images initialized via the image-dependent pair potential (IDPP) method. The FIRE optimizer was used with a force convergence criterion of fmax = 0.01 eV/A. The maximum number of optimization steps was set to 150 per path. All 35 paths converged successfully.

**SN3.4.1 Bulk Li Reference Barrier**

A bulk BCC lithium vacancy migration barrier was calculated as the reference:

E_bulk_(ML) = 0.0564 eV

This value is compared to the experimental bulk self-diffusion activation energy of E_a_ = 0.557 eV, confirming the systematic underestimation factor of ~10x typical for ML potentials.[9]

**SN3.4.2 Full NEB Barrier Results**

35 vacancy hop pathways were calculated. The complete results, sorted by forward barrier energy, are presented in Table S3.1. Pathways are classified as physically reasonable (barrier < 0.5 eV) or unphysical (barrier > 0.5 eV, typically involving long-range hops or collapsed NEB images).

**Table N1.** Complete ML-NEB barrier data for 35 vacancy migration pathways in Li grain boundaries

| Path ID | Sigma | Atom (i to f) | Hop Distance (A) | Barrier_fwd_ (eV) | Barrier_rev_ (eV) | NEB Steps | Physical |
| --- | --- | --- | --- | --- | --- | --- | --- |
| 33 | 9 | 123 to 51 | 2.559 | 0.0000 | 0.0054 | 7 | Yes (barrierless) |
| 43 | 9 | 87 to 15 | 2.628 | 0.0000 | 0.0093 | 5 | Yes (barrierless) |
| 1 | 3 | 23 to 36 | 2.689 | 0.0001 | 0.0000 | 7 | Yes |
| 39 | 9 | 140 to 96 | 2.649 | 0.0189 | 0.0000 | 8 | Yes |
| 36 | 9 | 51 to 7 | 2.649 | 0.0189 | 0.0000 | 8 | Yes |
| 4 | 3 | 12 to 8 | 2.776 | 0.0212 | 0.0000 | 10 | Yes |
| 2 | 3 | 23 to 7 | 2.776 | 0.0212 | 0.0000 | 10 | Yes |
| 6 | 3 | 47 to 31 | 2.776 | 0.0212 | 0.0000 | 10 | Yes |
| 5 | 3 | 36 to 32 | 2.776 | 0.0212 | 0.0000 | 10 | Yes |
| 7 | 3 | 32 to 37 | 2.945 | 0.0353 | 0.0000 | 13 | Yes |
| 8 | 3 | 7 to 22 | 2.945 | 0.0353 | 0.0000 | 13 | Yes |
| 51 | 9 | 87 to 7 | 5.682 | 0.0538 | 0.0000 | 150 | Marginal |
| 44 | 9 | 15 to 7 | 3.534 | 0.1979 | 0.0000 | 71 | Yes |
| 42 | 9 | 88 to 96 | 3.534 | 0.1979 | 0.0000 | 40 | Yes |
| 38 | 9 | 140 to 88 | 2.899 | 0.3889 | 0.0867 | 29 | Yes |
| 40 | 9 | 68 to 16 | 2.899 | 0.3889 | 0.0867 | 29 | Yes |
| 34 | 9 | 123 to 87 | 2.899 | 0.3889 | 0.0867 | 29 | Yes |
| 35 | 9 | 51 to 15 | 2.899 | 0.3889 | 0.0867 | 29 | Yes |
| 13 | 3 | 36 to 37 | 5.721 | 0.6706 | 0.6638 | 150 | No |
| 12 | 3 | 36 to 7 | 5.465 | 0.7094 | 0.7071 | 150 | No |
| 24 | 5 | 63 to 51 | 2.997 | 0.8084 | 0.7564 | 150 | No |
| 45 | 9 | 123 to 15 | 3.890 | 1.0646 | 0.7676 | 150 | No |
| 47 | 9 | 51 to 87 | 3.890 | 1.0660 | 0.7691 | 150 | No |
| 9 | 3 | 23 to 32 | 5.465 | 1.3080 | 1.3057 | 150 | No |
| 14 | 3 | 47 to 8 | 5.465 | 1.6969 | 1.6946 | 150 | No |
| 10 | 3 | 23 to 22 | 5.721 | 2.1769 | 2.1701 | 150 | No |
| 46 | 9 | 123 to 7 | 5.128 | 2.7782 | 2.7433 | 150 | No |
| 11 | 3 | 12 to 31 | 5.465 | 4.8037 | 4.8014 | 150 | No |
| 3 | 3 | 12 to 47 | 2.689 | 4.8344 | 4.8343 | 150 | No |
| 41 | 9 | 88 to 16 | 2.628 | 5.2547 | 5.2640 | 150 | No |
| 37 | 9 | 140 to 68 | 2.559 | 5.5662 | 5.5716 | 150 | No |
| 49 | 9 | 68 to 88 | 3.890 | 5.8062 | 5.5093 | 150 | No |
| 50 | 9 | 68 to 96 | 5.128 | 6.4987 | 6.4639 | 150 | No |
| 52 | 9 | 16 to 96 | 5.682 | 31.186 | 31.132 | 150 | No |
| 48 | 9 | 140 to 16 | 3.890 | 52.621 | 52.324 | 150 | No |

**SN3.5 NEB Calculations**

The ML-computed barriers were scaled to physically accurate values using the experimental bulk activation energy as a reference. The scaling factor alpha is defined as:

alpha = E_a_(exp) / E_bulk_(ML) = 0.557 eV / 0.0564 eV = 9.88

where E_a_(exp) = 0.557 eV is the experimentally measured bulk Li self-diffusion activation energy from Lodding et al..[9] The scaled GB barrier is then:

E_GB_(scaled) = alpha x E_GB_(ML)

This approach preserves the dimensionless ratio E_GB_/E_bulk_, which is insensitive to the systematic bias of the ML potential because the same model is used for both bulk and GB calculations.

**Table N2.** Representative GB minimum barriers and scaling results

| GB Type | Path ID | E_ML_ (eV) | Ratio (E_GB_/E_bulk_) | E_scaled_ (eV) | GB-enhanced? |
| --- | --- | --- | --- | --- | --- |
| Sigma3 | 4 | 0.0212 | 0.375 | 0.209 | Yes |
| Sigma9 | 39 | 0.0189 | 0.335 | 0.187 | Yes |
| Bulk | — | 0.0564 | 1.000 | 0.557 (exp) | (reference) |

Sigma3 and Sigma9 GBs exhibit barrier ratios significantly less than unity (0.375 and 0.335, respectively), indicating enhanced Li diffusion along these boundaries relative to the bulk. **SN3.6 NEB Calculations**

Using the Arrhenius relation D = D_0_·$e^{{-E_{a}}/{k_{B}}T}$ with D_0_ = 4.4 x 10^-5^ m^2^/s, the diffusion coefficients were calculated as a function of temperature: [9]

**Table N3.** Temperature-dependent diffusion coefficients

| T (K) | D_bulk_ (m^2^/s) | D_GB_, _Sigma3_ (m^2^/s) | D_GB_, _Sigma9_ (m^2^/s) | D_GB_, _eff_ (m^2^/s) |
| --- | --- | --- | --- | --- |
| 250 | 2.60e-16 | 2.69e-09 | 7.63e-09 | 4.53e-09 |
| 300 | 1.93e-14 | 1.35e-08 | 3.23e-08 | 2.09e-08 |
| 318 | 6.55e-14 | 2.14e-08 | 4.86e-08 | 3.23e-08 |
| 340 | 2.44e-13 | 3.51e-08 | 7.55e-08 | 5.15e-08 |
| 360 | 7.01e-13 | 5.21e-08 | 1.08e-07 | 7.49e-08 |
| 380 | 1.80e-12 | 7.43e-08 | 1.48e-07 | 1.05e-07 |
| 400 | 4.22e-12 | 1.02e-07 | 1.96e-07 | 1.42e-07 |

The effective GB diffusivity D_GB, eff_ is the weighted average of the GB-enhanced boundaries (Sigma3 and Sigma9). At the operating temperature of 318 K : - D_GB, eff_ = 3.23 x 10^-8^ m^2^/s - D_bulk_ = 6.55 x 10^-14^ m^2^/s - D_GB_/D_bulk_ = 4.93 x 10^5^ (~10^5.7^) This ratio of ~10^5.7^ falls within the expected range of 3-6 orders of magnitude for GB-to-bulk diffusivity enhancement.[10] The effective GB activation energy is:

E_a_ (GB,eff) = 0.198 eV

obtained from the weighted average of Sigma3 (E_a_ = 0.209 eV) and Sigma9 (E_a_ = 0.187 eV).

**SN3.7 NEB Calculations**

The Coble creep rate was calculated using the classical equation :[11]

epsilon_dot_ = (K * sigma * Omega * delta * D_GB_) / (k_B_ * T * D^3^)

where: - K = 148 (Coble creep constant for equiaxed grains) - sigma = applied stress (Pa) - Omega = atomic volume of Li = 2.18 x 10^-29^ m^3^ - delta = GB width = 5 x 10^-10^ m (0.5 nm, standard assumption) - D_GB_ = GB self-diffusion coefficient (m^2^/s) - k_B_ = Boltzmann constant = 1.381 x 10^-23^ J/K - T = temperature (K) - D = grain diameter (m)

**SN3.7.1 Grain Size Dependence at 318 K, 10 MPa**

**Table N4.** Coble creep rate as a function of grain diameter at T = 318 K, sigma = 10 MPa

| D (m) | D (label) | epsilon_dot__Coble (s^-1^) | log10(epsilon_dot_) |
| --- | --- | --- | --- |
| 5e-09 | 5 nm | 9.32e+08 | 8.97 |
| 1e-08 | 10 nm | 1.17e+08 | 8.07 |
| 2e-08 | 20 nm | 1.46e+07 | 7.16 |
| 5e-08 | 50 nm | 9.32e+05 | 5.97 |
| 1e-07 | 100 nm | 1.17e+05 | 5.07 |
| 2e-07 | 200 nm | 1.46e+04 | 4.16 |
| 5e-07 | 500 nm | 9.32e+02 | 2.97 |
| 1e-06 | 1 um | 1.17e+02 | 2.07 |

The D^-3^ dependence produces a factor of ~10^3.9^ between 5 nm and 100 nm grains, and ~10^6.9^ between 5 nm and 1 um grains. These ratios are D_0_-independent and demonstrate that nanoscale grain sizes dramatically enhance creep rates.

**SN3.7.2 Comparison of Creep Mechanisms**

Nabarro-Herring (lattice diffusion-mediated) creep rate:

epsilon_dot__NH = (28 * sigma * Omega * D_bulk_) / (k_B_ * T * D^2^)

Power-law (dislocation) creep rate:

epsilon_dot__PL = A * (sigma / G)^n^ * exp(-Q / k_B_*T)

where G = 4.2 GPa (shear modulus of Li), n ~ 4-5 (dislocation creep exponent), Q ~ 0.55 eV.

At 318 K and 10 MPa with D = 5 nm grains: - Coble creep rate >> Nabarro-Herring creep rate (by orders of magnitude) - The Coble-to-NH crossover grain size is approximately 2.6 mm, far exceeding any grain size in nanocrystalline lithium deposits. This confirms that Coble creep is the dominant deformation mechanism for lithium in the AFSSB operating regime.

**SN3.8 Comparison of Creep Mechanisms**

A Frost & Ashby deformation mechanism map was constructed with normalized stress (sigma/G) on the y-axis and homologous temperature (T/T_m_) on the x-axis,[12] where: - G = 4.2 GPa (shear modulus of Li at 300 K) - T_m_ = 453.65 K (melting point of Li)

The map delineates regions dominated by Coble creep, Nabarro-Herring creep, and power-law creep. The AFSSB operating point (T = 318 K = 0.70 T_m_, sigma = 10 MPa, sigma/G = 2.4 x 10^-3^) falls clearly within the Coble creep regime for grain sizes below ~100 um, confirming the mechanism proposed in the main text.

**SN3.9 Interface Energetics (ZnO Conversion Products)**

To address the role of ZnO conversion products (Li_2_O + LiZn) in facilitating ion transport across the interlayer, adsorption energies of a single Li atom on relevant surfaces were calculated using MACE-MP-0 :

**Table N5.** Li adsorption energies on interlayer surfaces

| Surface | E_ads (eV) | Interpretation |
| --- | --- | --- |
| Li_2_O (100) | -0.436 | Moderate affinity, ionic conductor |
| LiZn (110) | -0.988 | Strong affinity, electronic conductor |
| ZnO (10-10) | -15.7 | Extremely strong (chemical conversion) |
| Graphene | -1.390 | Reference (carbon host) |

The ZnO surface shows an extremely large E_ads_ (-15.7 eV) reflecting the thermodynamic driving force for the conversion reaction ZnO + xLi 🡪 Li_2_O + LiZn. Post-conversion, the Li_2_O and LiZn phases provide moderate-to-strong lithium affinity, supporting the mixed ionic-electronic conductor (MIEC) functionality. The Li migration barrier through Li2O was estimated from literature DFT values:

E_mig_(Li_2_O) = 0.31 eV

This low migration barrier confirms that Li_2_O, a major conversion product, does not impede Li-ion transport through the interlayer, addressing the reviewer concern about the effectiveness of the ZnO-derived interlayer in later cycles.

**SN3.10 Important Notes and Limitations**

1. D_0_ assumption: The pre-exponential factor D_0_ = 4.4 x 10^-5^ m^2^/s was used for both bulk and GB diffusion. This provides an upper-bound estimate for absolute creep rates. However, the grain size scaling exponent (D^-3^), the GB/bulk diffusivity ratio, and the dominance of Coble creep over other mechanisms in the deformation mechanism map are all independent of the D_0_ assumption.
2. ML potential systematic bias: MACE-MP-0 underestimates barrier heights by ~10x relative to experiment. The barrier ratio scaling approach corrects for this systematic bias by referencing all barriers to the experimental bulk activation energy.
3. GB type sampling: Three CSL GB types (Sigma3, Sigma9) were studied as representative boundaries in polycrystalline lithium. Real polycrystals contain a distribution of GB types; the effective GB diffusivity represents a weighted average dominated by the fastest pathways (Sigma3, Sigma9).

**Figure N6.** a) Li adsorption energies on interlayer surfaces b) Li diffusion (Arrhenius plot) c) Deformation mechanism map of lithium d) Coble Creep Rate vs. Grain Size

**Supplementary Figure**

**Figure S1**. DFT calculation of energy barrier, structural evolution and bonding preference for atoms in Zn nanoparticles with Li during lithiation

**Figure S2**. The cross-sectional SEM image after deposited lithium on the carbon layer after 0.3 mA cm^-2^ charging at 6 mAh cm^¬2^.

**Figure S3.** The EDS maps of zinc are shown in ZnO@C (e-beam) composite layer.

**Figure S4.** The EDS maps of zinc are shown in ZnO+C (mixing) composite layer.

**Figure S5.** The voltage profile on ZnO@C (e-beam) composite layer of 0–1.5 V at 0.3 mA cm^-2^.

**Figure S6.** Cross-sectional SEM image of plated Li after 10 cycles on the bare SUS.

**Figure S7.** Differential capacity plots (dQ/dV) during Li deposition on ZnO@C (e-beam) and ZnO+C (mixing) layer.

**Figure S8**. Li-Zn phase diagram

**Figure S9.** Cross-sectional SEM image of stripped Li on the ZnO@C (e-beam) composite layer.

**Figure S10.** a) XPS spectra of ZnO+C and ZnO@C for O 1s b) XPS spectra of ZnO+C for O 1s, c) XPS spectra of ZnO@C for O 1s.

**Figure S11.** Electrochemical properties of the ASSBs employing NMC9055-LPSCl composite cathode and Li metal, bare Ag+C (mixing) and ZnO@C (e-beam) layer anode. a) Initial voltage profiles for the ASSBs at 0.05 C. b) Cycle performances for 100 cycles at 0.1 C (1 C = 5.5 mA cm^−2^).

**Figure S12**. The Nyquist plots measured at room temperature and (inset) equivalent circuit of the argyrodite (Li_6_PS_5_Cl).

**Figure S13**. XRD patterns of the argyrodite (Li_6_PS_5_Cl).

**Figure S14**. Scheme of cell assembling containing a) cathode/SE/ZnO@C layer and b) Li metal/SE/ZnO@C layer.

**Table S1**. Model parameters for calculating the energy density of AFSSBs. Parameters marked in Table S1 are based on the previous study by *Lee et al.[13]

| Cell model parameters | | | |
| --- | --- | --- | --- |
|  | | **This work**  **(ZnO@C)** | 50 μm Li metal foil |
| Cathode | NMC 9055 capacity*  (mAh g^-1^) | 220 | |
|  | NMC 9055 ratio* (%) | 65 | |
|  | Thickness* (μm) | 100 | |
|  | Areal capacity*  (mAh cm^-2^) | 5.5 | |
| SSE | Thickness* (μm) | 30 | |
| Anode | Theoretically plated  Li thickness (μm) | 24.3 | 26.7 |
|  | ZnO@C composite layer thickness (μm) | 10 | - |
|  | Li metal thickness (μm) | - | 50 |
| Current collector | Al foil thickness (μm) | 12 | |
|  | SUS foil thickness (μm) | 10 | |
| Pouch | Packaging thickness* (μm) | 120 | |
|  | Insulating film thickness* (μm) | 40 | |
|  | Area* (cm^2^) | 57 (6.0 cm x 9.5 cm) | |
|  | Layer* | 10 | |
|  | Total thickness (μm) | 4220 | 5194 |
|  | Nominal voltage* (V) | 3.79 | |
|  | Cell capacity* (Ah) | 5.87 | |
|  | Energy density  (Wh L^-1^) | 925 | 752 |

**Table S2.** Cost comparison of Anode (Li metal/Ag-C/ZnO-C) materials and electrode including the electron-beam (e-beam) irradiation process

**Table S3.** Parameters used in relax calculation conducted by Quantum Espresso.

| **DFT setting** | | **Parameters** |
| --- | --- | --- |
| SCF convergence threshold | | 1.00 × 10^−6^ Ry |
| Energy convergence threshold | | 1.00 × 10^−4^ Ry |
| Force convergence threshold | | 2.00 × 10^−3^ Ry |
| Energy cutoffs of the plane wave | | 40 Ry |
| Energy cutoffs of the electron density | | 320 Ry |
| Hubbard parameters | Ag | 15.2 |
|  | Zn | 9.5 |

**Reference**

1.	D. Jun, J.-H. Lee, S. H. Park, S. Son, J. H. Lee, Y. J. Lee, *ACS Energy Letters* **2026**.

2.	N. Suzuki, N. Yashiro, S. Fujiki, R. Omoda, T. Shiratsuchi, T. Watanabe, Y. Aihara, *Advanced Energy and Sustainability Research* **2021**, 2, 2100066.

3.	S. H. Park, D. Jun, G. H. Lee, S. G. Lee, J. E. Jung, K. Y. Bae, S. Son, Y. J. Lee, *Advanced Science* **2022**, *9*, 2203130.

4.	D. Cao, T. Ji, Z. Wei, W. Liang, R. Bai, K. S. Burch, M. Geiwitz, H. Zhu, *Nano letters* **2023**, *23*, 9392-9398.

5.	Y. Wang, Y. Liu, M. Nguyen, J. Cho, N. Katyal, B. S. Vishnugopi, H. Hao, R. Fang, N. Wu, P. Liu, *Advanced Materials* **2023**, *35*, 2206762.

6.	J. Oh, S. H. Choi, J. Y. Kim, J. Lee, T. Lee, N. Lee, T. Lee, Y. Sohn, W. J. Chung, K. Y. Bae, *Advanced Energy Materials* **2023**, 13, 2301508.

7.	D. H. Kim, S. G. Kang, B. J. Kim, H. Lee, J. Kim, C.-B. Yoon, *Inorganics* **2023**, *11*, 440

8.	I. Batatia, P. Benner, Y. Chiang, A. M. Elena, D. P. Kovács, J. Riebesell, X. R. Advincula, M. Asta, M. Avaylon, W. J. Baldwin, *The Journal of chemical physics* **2025**, *163*.

9.	A. Lodding, J. Mundy, A. Ott, *physica status solidi (b)* **1970**, *38*, 559-569.

10.	J. S. Yoon, D. J. Siegel, *ACS Materials Letters* **2025**, *7*, 3581-3587.

11.	R. Coble, *Journal of applied physics* **1963**, *34*, 1679-1682.

12.	H. J. Frost, M. F. Ashby, *Oxford, Pergamon Press, 1982, 175 p* **1982**.

13.	Lee, Y.-G.; Fujiki, S.; Jung, C.; Suzuki, N.; Yashiro, N.; Omoda, R.; Ko, D.-S.; Shiratsuchi, T.; Sugimoto, T.; Ryu, S, *Nature Energy* **2020**, 5, 299-308.
